# 

**Published:** 1884-03-20

**Authors:** 


### *EDITORIAL NOTICES.*

THE STATE MEDICAL SOCIETY OF TENNESSEE meets in Chattanooga on Tuesday, April the 10th.

THE AMERICAN MEDICAL ASSOCIATION will meet in Washington City on May 1st, 1884.

THE TEXAS MEDICAL ASSOCIATION meets at Belton on the 22d of April next. The doors of the citizens are to be thrown open to the delegates and to their wives. Well done, Belton.

THE INTERNATIONAL MEDICAL CONGRESS meets at Copenhagen August 10th, 1884.

The British Medical Association meets at Belfast July 29th next.

Parties visiting these Associations can take Cunard Steamer which leaves New York July 16th. Steamers also leave on 12th and 15th of July.

MEDICAL ASSOCIATION OF GEORGIA.—We received the circular invitation of the Committee of Arrangements to attend the Medical Association of the State, which is appointed for the 16th of April at Macon, Georgia. The kind and cordial tone of the invitation, in connection with the well known generous hospitality of the profession at Macon, will be likely to elicit a large representation. We trust that such will be the case, and that it will be a reunion of medical brethren long to be remembered. We hope that the proceedings will be characterized by that broad and liberal spirit which will tend to harmony and to good feeling among the entire profession of the State.

BEAUTIFUL DRUG CASE.—The most beautiful pocket Drug Case we have ever seen was one furnished by the celebrated Drug House of McKesson & Robbins, and presented at the late Commencement Exercises by the Professor of Physiology in the Southern Medical College to Dr. Wm. R. McCrary for the best examination in the Department of Physiology. The case was not only beautiful, but most convenient and admirably gotten up, and filled, and labeled, with a great variety of those unique and exquisitely made gelatine coated pills and granules for which the House of McMesson & Robbins is justly celebrated.

ANNOUNCEMENT CONCERNING PRIZE ESSAYS ON THE SUBJECT OF CANCER.—In March, 1882, a prize of One Thousand Dollars was offered for the best Essay—if thought worthy of the prize—on the following subject: "Probability of the Discovery of a Cure of Malignant Disease, and the Line of Study or Experimentation likely to Bring such a Cure to Light." And the undersigned was delegated to receive essays until December 1st, 1883, and, with such assistance as he should select, to decide upon their merits.

The undersigned hereby announces that six essays on the above subject were presented to him, and that, in the consideration of their merit, the aid of Dr. George B. Shattuck, Editor of the Boston Medical and Surgical Journal, was enlisted. The result of this consideration is that no essay was deemed worthy of the prize. J. COLLINS WARREN, M.D.. M.M.S.S.,

January 1, 1881. 58 Beacon St., Boston, Mass.

One of the contestants for the above prize thinks that he was justly entitled to the prize, as no test was made of his method of cure by the Committee. He has promised to send the essay for publication in our Journal.

SARCO PEPTONES.—This article, a sample of which has been kindly sent us by that excellent house, Parke, Davis & Co., of Detroit, strikes us with great favor as a Food Preparation. It is an extract of Peptonized beef, and being Peptonized or digested already, is said to be most easily assimilated by the most delicate stomach. See the advertisement of this excellent preparation in this number of the Record.

### *RECEIPTED.*

1884.—Dr T L Turk, Dr Redding, H Nance to Oct., A B McWhorter, J J Stephens, J R Johnson, E Wheeler, R E Toombs, W B Thompson, E M Pharr to Oct., R MMcCants, James Watts, Robert A Miller, Wily Morgan, P W Butler, H L Sutherland, John Elsnor, E H Wright, E W Hawkins, W M Coleman, E H M Parham, Z T Young, J C Smith, J M Suttle, T T Logan, Thos Whiteman, R S Sampson, William Toon.

1883.—W C Moore, C A Jordan, R D Lucius, R J McMullen, R L Seal to Oct., R H Edwards.

1885.—A G Grove, P A Sloan, Morgan Callahan.

